# Perspectives of speech-language therapists on patient safety incidents in South Africa

**DOI:** 10.4102/safp.v67i1.6071

**Published:** 2025-02-21

**Authors:** Ntandoyenkosi L. Msomi, Suvishka Barath, Andrew J. Ross

**Affiliations:** 1 Department of Family Medicine, College of Health Sciences, University of KwaZulu-Natal, Durban, South Africa

**Keywords:** speech-language therapist, patient safety incident, thematic analysis, rehabilitation professions education, South Africa

## Abstract

**Background:**

Patient safety incidents (PSIs) cause harm to patients, including falls, accidental ingestion and physical assault. Despite their importance in healthcare, limited information exists on how South African speech-language therapists (SLTs) perceive them in the public and private sectors. This study applied the Donabedian model of patient safety and healthcare quality to SLTs’ perspectives on PSIs.

**Methods:**

Free attitude interviews with 10 South African SLTs explored the environment (where and who), processes (how and why) and outcomes (events and consequences) of PSIs. Interviews were transcribed and analysed thematically using a six-phase deductive and inductive approach within the Donabedian model.

**Results:**

The Donabedian model’s three components (structure, process, outcome) led to six sub-themes, highlighting its applicability to SLTs’ perspectives on PSIs. It revealed how factors such as clinical environment, care delivery and patient outcomes influence SLTs’ perceptions.

**Conclusion:**

Understanding SLTs’ perspectives is essential for addressing environmental issues, developing training, institutional inductions and audits that prevent and manage PSIs, and improving service quality.

**Contribution:**

This study emphasises engaging practitioners to understand factors affecting PSIs. It contributes to improving SLT training and practice in South Africa to enhance patient safety.

## Introduciton

Patient safety is commonly understood as freedom from any harm that is associated with healthcare service provision,^1^ with patient safety incidents (PSIs) being events that could have resulted, or did result, in unnecessary harm to a patient. Globally, PSIs are regarded as an indicator of healthcare quality, a priority in policy development, and an opportunity to improve healthcare systems holistically.^2^

In 2002, the World Health Organization (WHO) convened a summit because of the alarming rates of preventable patient deaths and safety incidents to develop plans to strengthen health systems.^3^ Most health ministries in the global north and the Middle East who were in attendance signed declarations to acknowledge medical errors and adverse events and endeavoured to pilot and implement patient safety protocols globally.

Since then, the rates of PSIs are closely monitored with low- and middle-income countries statistics showing 134 million adverse events contributing to 2.6 million deaths annually.^4^ However, being a signatory is voluntary, with countries being left to decide how to address their PSI rates. In the United Kingdom, 100 000 monthly PSIs were reported in 2018, highlighting the need for interventional programmes to decrease this rate.^5,6^ Patient safety incidents also continue to be reported in South Africa in the public sector, the Department of health spending R68 billion in March 2023 in compensation for patient injuries and medical negligence.^7^ By law, PSIs that occur in all health disciplines are required to be reported to the PSI register, where they are physically or electronically captured and analysed.^8^ A standard reporting form is available through the National Department of Health website that can be downloaded. This enables health facilities to monitor the number of PSIs.^9^

The WHO developed the International Classification of Patient Safety to enable comparisons of global PSI statistics in 2009, which consists of five degrees of PSI severity that start from no harm to mild, moderate, severe, and eventually, death.^10^ The Health Professions Council of South Africa (HPCSA) is a statutory body comprising 12 professional bodies that govern the education, training and registration of healthcare professionals in South Africa. Speech-language therapists (SLTs) belong to this body and have specialised expertise in managing communication, eating, hearing, language, literacy, speech and swallowing disorders.^11,12^ The most common sources of PSIs that are relevant to SLTs include diagnostic errors, patient falls and problem misidentification. However, PSIs typically involve numerous aspects that are interrelated and have the potential to cause harm to a patient. Factors within systems and organisations, such as interruptions in workflow and care coordination, limited resources, insufficient staffing, and challenges in competency enhancement, can contribute to the incidence of PSIs.^13^

Factors that are associated with human behaviour, such as communication breakdown among healthcare professionals, patients and their families, as well as ineffective multidisciplinary collaboration, fatigue and burnout, can also result in PSIs.^14^ Patient-related factors include limited health literacy, a lack of engagement during therapy sessions, and non-adherence to the use of assistive devices, while technological factors, such as uncalibrated equipment, can also cause PSIs.^15^ Understanding the perspectives of SLTs regarding PSIs is essential for promoting a culture of safety and reducing the risk of harm to patients. By addressing these factors and embracing a proactive approach to patient safety, SLTs can contribute to an improved quality of care and better outcomes for individuals who access healthcare services. This study aimed to apply the Donabedian model of patient safety and healthcare provision quality to SLTs’ perspectives on PSIs.

## Conceptual framework

The Donabedian model of patient safety and healthcare provision quality was regarded as an appropriate framework to achieving the aim of the study, as it recognises the three components of structure, process and outcome^16^:

- ***Structure***: This denotes the environment where a healthcare service is provided and its components, such as the health system, healthcare professionals, and the patient. In the context of this study, the environment is understood to be public and private healthcare institutions and special needs schools.^17^
- ***Process***: This involves the interactions among healthcare professionals, and between healthcare professionals and patients, which can be physical or technical.^17^ In this study, this includes the professional training of SLTs, and the PSIs identified and reported in various settings.
- ***Outcome***: This includes the prognosis or treatment of patients, their quality of life, satisfaction with the healthcare services provided, and consequences of clinical services.^17^

The three components ensure patient safety and high-quality healthcare. According to this framework, the environment in which care is delivered may affect how well it is provided, thereby raising or lowering the standard of care and the outcomes. While modifications to the process may affect the process itself, the outcomes are influenced by the structures and processes, with modifications in either one possibly affecting the patient’s health.

## Research methods and design

The study used participatory action research, which consisted of various qualitative methods that aimed to share co-produced stakeholder information and education materials on PSIs. The main study consisted of three phases: (1) free attitude interviews, (2) two asynchronous text-based focus groups, and (3) artifact analysis. The initial phase was conducted using a descriptive interpretative approach. This approach was appropriate for exploring the what, who, and where of PSIs, and extrapolating participants’ insights into this phenomenon.^18^ Participant welfare and confidentiality were ensured through obtaining written informed consent, removal of personal identifiers and using codes, and password protected storage of data for 3 years.^19^

Free virtual attitude interviews were conducted with 10 SLTs. This format was regarded as appropriate for logistical reasons, such as reduced travel costs, the broad participant population, and convenience without compromising the collected data’s quality.^20^

### Participants

Participants were selected intentionally from a network of SLTs’ professional journal clubs and were required to be registered with the HPCSA as independent practitioners who had experience working with people in the special education, private and public healthcare sectors. An attempt was made to exclude those who were dually qualified practitioners as audiologists and SLTs. Of the 20 SLTs who were contacted via an email and invited to participate in this study, having been provided with detailed information letter and a consent form, 10 returned signed consent forms and participated. The use of 10 SLTs as participants in this study was guided by logistical feasibility and convenience. Furthermore, the study adhered to qualitative sample size determination principles, particularly the concept of ‘information power’, as outlined by Malterud and colleagues.^21^ This approach suggests that the adequacy of sample size in qualitative research is contingent on factors such as the study aim’s specificity, the sample’s specificity, and the quality of the collective dialogue. Saturation was used as a key criterion during data collection. As data analysis progressed, the researchers observed that no new themes or significant insights emerged after interviewing the 9th and 10th participants, indicating that data saturation had been achieved. This ensured the robustness and credibility of the study findings while maintaining alignment with qualitative research standards.

### Materials

A free attitude interview protocol was developed as guided by the steps of the Interview Protocol Refinement (IPR) Framework.^22^ The development included: (1) aligning the interview questions with the research question; (2) conducting an inquiry-based discussion; (3) gathering feedback from co-investigators about the interview protocol; and (4) piloting the interview protocol. The main interview questions were conceptualised according to the three components of the Donabedian model of patient safety and healthcare provision quality.^16^ Feedback was received from one expert with a background and experience in healthcare research, and the interview protocol was refined further.

### Procedure

The interviews were hosted on the Teams platform.^23^ Each participant was assigned a code before being admitted to the platform, as described in the study information letter. Participants’ demographic information, such as age, the university they graduated from and years’ working experience, were collected before the main questions were introduced. The interviews lasted for 40 min and were recorded by Microsoft Teams for later transcriptions.

### Data analysis

The Teams interview transcripts were downloaded and checked against audio recordings, then uploaded onto NVivo for thematical analysis. An embedded codebook approach of thematic analysis, as described by Braun and Clarke, was used to analyse the qualitative data. This approach was appropriate for research on health professions that aims to guide clinical practice.^24^ A combination of deductive and inductive coding was used, with the main preconceived themes relating to the three components of the Donabedian model of structure, process and outcomes, while the codes and sub-themes were deduced inductively from the data. Inductive coding focuses on semantic meanings, rather than on coding the latest meanings. The first author read the transcripts three times and annotated them with notes, with data related to the overall aim being coded in the first coding cycle. Codes were then grouped into sub-themes and aligned with the research questions, with a provisional codebook being developed. The second author independently followed the same data analysis steps, after which the coding was refined through reflective discussions between them, which strengthened the credibility of the study’s findings.^25^

### Researchers

The first author, who conducted the interviews, is a trained and practising South African SLT and lactation consultant who works at a public sector regional hospital in KwaZulu-Natal Province and has 3 years’ experience in clinical and leadership roles. The second author is a South African audiologist who works in a private hospital, has 3 years’ experience and is pursuing doctoral studies at a local university. The third author is a family physician, with teaching expertise on PSI at a local medical school.

### Ethical considerations

Ethical clearance to conduct this study was obtained from the University of KwaZulu-Natal Humanities and Social Sciences Research Ethics Committee (No. HSSREC/00007277/2024).

## Results

Of the 20 participants invited, 10 agreed to participate. Among them, 7 were females aged 24 to 27 years. Three worked in the public health sector, 4 in private healthcare, and 3 in special education, which falls under the public sector. Two participants had obtained an MSc in public health from a local university as a second qualification (Table 1).

**TABLE 1:** Participants’ demographics.

Code	Age	Gender	Sector	Years’ experience
P1	24	Female	Private Healthcare	2
P2	24	Female	Public Healthcare	2
P3	24	Female	Public Education	2
P4	28	Female	Public Education	4
P5	26	Male	Public Healthcare	3
P6	25	Female	Public Healthcare	3
P7	26	Male	Private Healthcare	2
P8	27	Male	Private Healthcare	3
P9	24	Female	Public Education	2
P10	27	Female	Private Healthcare	3

Table 2 presents the themes that emerged from analysing the data with respect to the three aspects of the conceptual framework and the associated sub-themes. To ensure anonymity, each participant was allocated a unique code, and their details regarding age, gender, sector and years’ experience indicated.

**TABLE 2:** Themes and sub-themes from the data.

Themes	Sub-themes
1. Structure	1.1. Describing PSI 1.2. Environmental risk factors
2. Process	2.1. Training insights 2.2. PSI encounters – observations from the environment 2.3. Reporting PSIs
3. Outcomes	3.1. Technological advances: perspectives on change

PSI, patient safety incidents.

The participants’ perspectives on PSIs yielded several critical insights, which will be discussed in detail in the subsequent sections.

### Theme 1: Structure

Structure relates to where a healthcare service is provided and its components, such as the health system, healthcare professionals, and the patient, with two sub-themes emerging: *describing patient safety incidents*; and *environmental risk factors*.

#### Sub-theme 1.1: Describing patient safety incidents

Participants provided various descriptions of PSIs in both the public and private sectors, highlighting their understanding of needing to be aware of the risks that their patients may be exposed to, both intentional and unintentional, which may affect service provision:

‘… any hazards that might pose a risk to the safety of patients while [*they are*] in our care.’ (P2, 24 years old, Public Healthcare)

‘Patient safety incidents may happen with the patient while they are under your care or your supervision that intentionally, [*or*] unintentionally, might end up harming them or affecting their health in terms of their condition and diagnosis.’ (P9, 24 years old, Public Education)

Some participants also included non-physical aspects of patient–clinician interactions, such as ensuring that their rights to privacy are protected:

‘It is broad; I think of PSI[*s*] is keeping patient information confidential to protect their privacy.’ (P3, 24 years old, Public Education)

#### Sub-theme 1.2: Environmental risk factors

Participants highlighted various factors that contributed to PSIs in their respective environments, including the characteristics of the populations they serve and mentioned how infrastructural elements can pose risks:

‘We work with many children who are on the spectrum or have ADHD, so there is much running around.’ (P3, 24 years old, Public Education)

Another participant noticed that sharing a space in clinics with other professions increased environmental risk, stating the following:

‘We are at a clinic, so we share a common space with OT [*occupational therapy*]; the equipment and toys overstimulate tactile-seeking children. A PSI can happen at any time.’ (P6, 26 years old, Public Healthcare)

‘There is no child-friendly waiting area. When children with behavioural issues wait for long in these areas, it is a problem.’ (P5, 26 years old, Public Healthcare)

### Theme 2: Process

Process relates to interactions among healthcare professionals, and between healthcare professionals and patients, which can be physical or technical, with three sub-themes emerging, these being training insights, PSI encounters and reporting PSIs.

#### Sub-theme 2.1: Training insights

All participants discussed the lack of training received regarding PSIs during their undergraduate education, with some having their first exposure once they started working:

‘Patient safety incidents were not mentioned in school, but it is something that you face each and every single day. So, they could have focused on that so much more.’ (P3, 24 years old, Public Education)

‘We had disorder-specific protocols in university, but I do not think the protocols had information about preventing PSI.’ (P10, 27 years old, Private Healthcare)

‘The first time I heard about PSI was during a quality assurance workshop in my community service year.’ (P7, 26 years old, Private Healthcare)

Most participants expressed a need for training on PSIs, in relation to their scopes of practice in dealing with patients whose conditions posed specific risks:

‘We would benefit from additional training on PSI, since we manage feeding and swallowing disorders.’ (P1, 24 years old, Private Healthcare)

#### Sub-theme 2.2: Patient safety incident encounters – observations from the environment

Participants recounted their experiences with PSIs associated with their workplaces, including the handling of what should have been confidential patient reports, and the risks associated with patient management incidents that they had experienced:

‘So, we had to do a home visit for this assessment. So, I remember we did the assessment. We finished. I took the forms and remembered putting them at the back of the car; they were not even upside down. They were facing the top, and we left the car. We went to see other children.’ (P3, 24 years old, Public Education)

‘An incident happened, but it had nothing to do with my professional speech. However, in present practice, we are required to walk our patients to the therapy room, and this patient was not wearing appropriate shoes. The patient was wearing socks that were gripped, and the patient was also aggressive, so the patient did not want to follow the walking precautions. I have to walk with the patient, hand in hand, and hold the rail. The patient refused to walk hand in hand.’ (P6, 25 years old, Public Healthcare)

#### Sub-theme 2.3: Reporting patient safety incidents

Participants shared insights into the instruments that were used to report and identify PSIs within their institutions and found various reporting measures that were taken to ensure timeous report delivery:

‘We mainly use our SOAP [*subjective, objective assessment, and plan*] notes as the biggest thing. So, you write in the SOAP notes exactly what happened, exactly what occurred, and the first contact person you go to is the manager.’ (P1, 24 years old, Private Healthcare)

‘So, it would be an incident book. Incidences are recorded there, and it [*the incident book*] is in an accessible place, where everyone can read them [*incidences*].’ (P9, 24 years old, Public Education)

A participant from the public healthcare sector elaborated as follows:

‘So, this is the PSI form that you fill in, that would have its details, and then you write what your name [is] and how many things happened, and then you also write what steps did you take [*sic*] to address the issue.’ (P5, 26 years old, Public Healthcare)

### Theme 3: Outcome

This theme relates to the outcomes associated with consequences of service provision and quality of life, and consisting of one sub-theme: technological advances – perspectives on change.

#### Sub-theme 3.1: Technological advances – perspectives on change

Across the sectors, participants expressed thoughts on how technological advances could positively and negatively affect PSIs in speech-language therapy, with some observing that the Department of Education may not keep pace with technological advances that could benefit the learners. Participant 4 felt that the Department of Education (DoE), in its role as a service provider, could integrate technological advances in the education sector:

‘Oh, if I look at South Africa specifically, there is [*a*] potential that we could get to that level.’ (P4, 28 years old, Public Education)

Others expressed concerns that technological advancements, such as artificial intelligence (AI), might undermine the humanistic aspect of speech-language therapy:

‘It is not something I have thought of, but the introduction of AI is something that, I fear. As speech therapists, our scope of practice and profession is very people based. Thus, the introduction of AI takes away that empathy. Engagement that [*or through which*] rapport is built, and I think it [*the introduction of AI*] may increase adverse incidents, including PSIs.’ (P8, 27 years old, Private Healthcare)

## Discussion

The participants’ descriptions of PSIs encompassed both tangible and intangible aspects of patient care. The varied descriptions underscored the complexity of PSIs, suggesting that SLTs perceive these incidents as not only direct physical harm but also related to confidentiality and the integrity of patient–clinician interactions. This broad understanding aligns with the literature that emphasises the importance of considering the psycho-social dimensions of patient safety, particularly in rehabilitation settings, where communication plays a pivotal role.^26^ Issues related to environmental risk factors included how the physical and social characteristics of rehabilitation settings contribute to the occurrence of PSIs. Participants highlighted the challenges of working in spaces that they shared with other disciplines, and the implications of specific client population’s needs, such as children with pervasive developmental disorders. Over-stimulating environments and the absence of child-friendly waiting areas can exacerbate behavioural issues, potentially leading to incidents that compromise patients’ safety. This finding resonates with studies that identified environmental factors as contributors to patient safety risks.^27^

Insights into training revealed a gap in the education and preparation of SLTs regarding PSIs, which their formal education did not adequately address, thereby indicating a need for curriculum reforms to prioritise patient safety instruction. This lack of emphasis on PSIs during academic training is concerning, as it limits SLTs’ preparedness to anticipate, prevent and manage these incidents effectively in their practice.^4^ Ongoing professional development and targeted training workshops could bridge this gap by equipping therapists with the necessary skills to recognise and mitigate PSIs proactively. In examining the processes related to PSIs, participants shared first-hand accounts of their experiences and highlighted the importance of situational awareness and observational skills in preventing incidents. The stories of missed documentation during assessments and unsafe patient handling underscore the important role of attention to detail in practice to prevent PSIs from occurring. These findings suggest that enhancing communication among multidisciplinary teams, as well as improved documentation practices may reduce the risk of PSIs in therapeutic settings.^5^

The participants emphasised the importance of a systematic approach to reporting PSIs, detailing the processes that were in place within their organisations. Although some reported using SOAP notes (a structured method of documentation covering Subjective details, Objective findings, Assessment, and Plan) and incident books, the effectiveness of PSI reporting mechanisms depends on the organisational culture surrounding patient safety. Open communication and regular feedback are essential for fostering a culture that encourages the reporting and discussion of PSIs without fear of retribution.^6^ Institutional support in implementing transparent reporting systems and regular training on these protocols are therefore essential for promoting patient safety. The discussions surrounding outcomes revealed divergent perspectives on the impact of technological advancements on PSIs in speech-language therapy. While some participants acknowledged the potential benefits of technology in streamlining processes and improving patient engagement, others raised concerns about the depersonalisation of care through the introduction of AI. Their concerns related to the automation of testing and treatment provision, with SLTs no longer being needed to interact with patients, and their role being reduced to administrative tasks. This apprehension aligns with research suggesting that although technology can enhance efficiency, it may also detract from the empathetic and relational aspects of therapeutic practice that requires therapist to listen to and communicate with their patient.^28^ Therefore, balancing technological integration with the preservation of humanistic care is essential in mitigating the risk of PSIs arising from reduced clinician–patient rapport.

### Limitations

Speech-language therapists who agreed to participate in the study may have been more likely to be professionally interested in PSI, which could introduce a degree of participant bias. As this study is qualitative and has such a small sample size, generalisations of its findings to the broader South African SLT population should be made cautiously.

## Conclusion

This study’s findings provide valuable insights into SLTs’ perceptions of PSIs in South Africa. By addressing the structural, process-orientated, and outcome-related dimensions of patient safety, healthcare institutions can develop targeted strategies to improve safety practices and enhance the overall quality of care in speech-language therapy settings.

### Recommendations

Further studies are needed to advance SLTs’ understanding of PSIs and improve their prevention, management and mitigation practices. Deliberation between researchers and practitioners is needed for them to agree on an acceptable definition of a PSI, specifically in the field of speech-language therapy, but in rehabilitation professions in general, where professionals also work in non-medical settings, such as schools, often as part of multidisciplinary teams. While SLTs’ primary purpose is to improve the quality of life of the patients they are providing services to, they also need to ensure that they are not harmed during the process. Providing holistic care needs to ensure that the environment in which the services are provided does not compromise their health or rights in any way, that the processes they undergo are rigorous, and that the outcomes are favourable.

## References

[1] World Health Organization. Exploring patient participation in reducing health-care-related safety risks. 2013.

[2] Snowdon A, Hussein A, Danforth M, Wright A, Oakes R. Digital maturity as a predictor of quality and safety outcomes in US hospitals: Cross-sectional observational study. J Med Internet Res. 2024;26:e56316. 10.2196/56316 39106100 PMC11336495

[3] WHA55 R. 18. Quality of care: Patient safety. Fifty-fifth World Health Assembly. Geneva: World Health Organization, 2002; p. 18.

[4] Khawagi WY. Assessing the safety of prescribing for people with mental illness through the development and implementation of prescribing safety indicators. Manchester: The University of Manchester (United Kingdom); 2021.

[5] Berwick D. A promise to learn-a commitment to act: Improving the safety of patients in England. United Kingdom: Department of Health; 2013.

[6] Cooper J, Williams H, Hibbert P, et al. Classification of patient-safety incidents in primary care. Bull World Health Organ. 2018;96(7):498. 10.2471/BLT.17.199802 29962552 PMC6022620

[7] Khan M, Gardner J. Clicks, carelessness and consequences: Navigating pharmacist negligence. S Afr J Bioeth Law. 2024;17(3):e2336. 10.7196/SAJBL.2024.v17i3.2336

[8] Gqaleni T, Mkhize S. Healthcare professionals’ perception of knowledge and implementation of Patient Safety Incident Reporting and Learning guidelines in specialised care units, KwaZulu-Natal. South Afr J Crit Care. 2023;39(1):25–29. 10.7196/SAJCC.2023.v39i1.559 PMC10378188 37521960

[9] Gqaleni TM, Mkhize SW, Chironda G. Patient safety incident reporting and learning guidelines implemented by health care professionals in specialized care units: Scoping review. J Med Internet Res. 2024;26:e48580. 10.2196/48580 39365987 PMC11489802

[10] World Health Organization. Conceptual framework for the international classification for patient safety. Version 1.1. Final Technical Report. 2009.

[11] Antoni C. The role of the speech and language therapist. Transsexual and other disorders of gender identity. CRC Press, 2017; p. 137–156.

[12] Du Plessis S. Exploring the role of the speech-language therapist in city centre preschools. S Afr J Child Educ. 2012;2(2):23. 10.4102/sajce.v2i2.14

[13] Safety WP. Global priorities for patient safety research. 2009. 10.1136/bmj.b1775 19443552

[14] Wahr JA, Prager RL, Abernathy Iii J, et al. Patient safety in the cardiac operating room: Human factors and teamwork: A scientific statement from the American Heart Association. Circulation. 2013;128(10):1139–1169. 10.1161/CIR.0b013e3182a38efa 23918255

[15] Vincent C. Patient safety. Chichester, John Wiley & Sons; 2011.

[16] Braithwaite J, Donaldson L. Patient safety and quality. In: Raithwaite J, Donaldson L, editors. The Oxford handbook of health care management. Oxford: Oxford University Press, 2016; p. 325–351.

[17] Voyce J, Gouveia MJ, Medinas MA, Santos AS, Ferreira RF. A Donabedian model of the quality of nursing care from nurses’ perspectives in a portuguese hospital: A pilot study. J Nurs Meas. 2015;23(3):474–484. 10.1891/1061-3749.23.3.474 26673771

[18] Ponelis SR. Using interpretive qualitative case studies for exploratory research in doctoral studies: A case of information systems research in small and medium enterprises. Int J Dr Stud. 2015;10:535. 10.28945/2339

[19] Pietilä A-M, Nurmi S-M, Halkoaho A, Kyngäs H. Qualitative research: Ethical considerations. In: Kyngäs H, editor. The application of content analysis in nursing science research. Cham: Springer, 2020; p. 49–69.

[20] Oliffe JL, Kelly MT, Gonzalez Montaner G, Yu Ko WF. Zoom interviews: Benefits and concessions. Int J Qual Methods. 2021;20:16094069211053522. 10.1177/16094069211053522

[21] Malterud K, Siersma VD, Guassora AD. Sample size in qualitative interview studies: Guided by information power. Qual Health Res. 2016;26(13):1753–1760. 10.1177/1049732315617444 26613970

[22] Castillo-Montoya M. Preparing for interview research: The interview protocol refinement framework. Qual Rep. 2016;21(5):811–831. 10.46743/2160-3715/2016.2337

[23] Ilag BN. Introducing Microsoft teams. Understanding the new chat-based workspace in office. Volume 365. 2018; p. 1–2.

[24] Terry G, Hayfield N, Clarke V, Braun V. Thematic analysis. In: The SAGE handbook of qualitative research in psychology. 2nd edn., London: SAGE Publications, 2017; p. 17–37.

[25] Bans-Akutey A, Tiimub BM. Triangulation in research. Acad Lett. 2021;2:1–6. 10.20935/AL3392

[26] Carayon P, Karsh B-T, Gurses AP, et al. Macroergonomics in health care quality and patient safety. Rev Hum Factors Ergon. 2013;8(1):4–54. 10.1177/1557234X13492976 24729777 PMC3981462

[27] Wears R, Sutcliffe K. Still not safe: Patient safety and the middle-managing of American medicine. New York, NY: Oxford University Press; 2019.

[28] Osborne C. What is the client experience of online video-calling equine-facilitated therapy? A heuristic study. 2023.

